# Traditional Healers as Health Care Providers for the Latine Community in the United States, a Systematic Review

**DOI:** 10.1089/heq.2021.0099

**Published:** 2022-06-15

**Authors:** Maria L. Cruz, Samantha Christie, Estrella Allen, Erika Meza, Anna María Nápoles, Kala M. Mehta

**Affiliations:** ^1^ Department of Public Health, San Francisco State University, San Francisco, California, USA.; ^2^ Department of Biology, San Francisco State University, San Francisco, California, USA.; ^3^ Department of Epidemiology and Biostatistics, University of California, San Francisco, California, USA.; ^4^ Division of Intramural Research, National Institute on Minority Health and Health Disparities, Bethesda, Maryland, USA.

**Keywords:** Latine, traditional healers, *curanderos*, *sobadores*, *yerberos*, *espiritualistas*

## Abstract

**Background:**

Due to structural barriers to accessing the biomedical health care system, traditional healers (THs) often serve as the first point of contact for health care by Latine individuals in the United States. A recent assessment of the extent of use of THs by the Latine community is lacking.

**Methods:**

We conducted a systematic review of the literature published between 2000 and 2020, to assess the prevalence of use of THs by U.S. Latine individuals, health conditions for which care was sought, reasons for their use, and extent of TH use and dual use that is of biomedical health care and TH together. Primary inclusion criteria for studies included: (1) published in English, (2) focus on THs, (3) pertained to Latine individuals residing in the United States, and (4) published since 2000.

**Results:**

Eighty-five studies were reviewed; 33 met inclusion criteria. Under the overarching term of *curanderos*, 4 subtypes of THs were identified: *sobadores*, *yerberos*, *espiritualistas*, and *hueseros*. The lifetime prevalence of TH use varied from 6% to 67.7% depending on the demographic differences among the Latine individuals in these studies. Primary reasons for seeking care from THs were accessibility/convenience, affordability, and linguistic and cultural congruence.

**Discussion:**

The use of THs is highly prevalent for Latine community residing in the United States because they are accessible, affordable, and provide culturally and linguistically compatible care, indicating that they offer an alternative that addresses systemic structural barriers to biomedical health care. Further research on the efficacy and safety of the treatments rendered by THs and how their care might be optimally coordinated with biomedical health care, could improve health equity and access to care among Latine individuals in the United States.

## Introduction

In July 2019, Latine individuals represented 19% (60.6 million) of the total U.S. population^1,2^ and accounted for more than half of the population growth in the United States between 2010 and 2019.^3^ We use the term Latine individuals to describe people of Latin American or Hispanic origin because it is a gender-neutral form of the word Latino.

Latine individuals have disproportionately higher rates of obesity, diabetes, uncontrolled high blood pressure, heart disease, and cancer compared with non-Latine Whites.^4–6^ Many systemic structural factors in the United States have led to Latine individuals' lower socioeconomic status, on average, compared with the non-Latine White population.^7^ Historically, Latine individuals in the United States have also faced large disparities in health insurance coverage.

In the United States, health care services are provided mainly through employer-based health insurance, Medicare, or Medicaid, and individuals not covered under these may face barriers (e.g., immigration status, low-wage paying jobs) to seek and pay for health care services.^8^ As a result of barriers to accessing the biomedical health care system, the Latine community, especially immigrants, seek complementary care, including those provided by traditional healers (THs). Understanding the role of THs in the Latine community may inform ways to combine traditional healing with biomedical health care to promote health equity for Latine individuals in the United States.

THs residing in the Latine community can serve as the first point of care for Latine individuals and their families who do not have access to other medical care, or as an alternative for Latine individuals who may be dissatisfied with biomedical health care because biomedical providers may not be able to deliver linguistically and culturally appropriate care.^9^ For example, THs are often sought because they speak a community member's native indigenous language or dialect (e.g., Quechua, Aymara, Portuguese, and Guatemalan dialects like Quiché, Mam, Pocomam, Chol, Carib).^10^ Moreover, these THs engender trust as they retain traditional, indigenous belief systems about health, and use these beliefs and systems to serve the Latine community in a way that is familiar to them.

The World Health Organization defines traditional medicine as the “sum total of the knowledge, skills, and practices based on the theories, beliefs, and experiences indigenous to different cultures, whether explicable or not, used in the maintenance of health as well as in the prevention, diagnosis, improvement, or treatment of physical and mental illness.”^11^ There are many types of THs and services that the Latine community utilizes in the U.S. Consistent with the literature and practice, we will use the term *curandero* (means “someone who heals” in Spanish) to refer to THs in this review.^10,12,13^ Curanderos can be classified into four major subtypes: *sobadores*,^9,14,15^ *yerberos*,^14^ *espiritualistas*,^16^ and *hueseros*,^14^ who use diverse approaches to heal patients. Sobadores often perform massage-like therapy and commonly treat “empacho” or constipation and musculoskeletal pain.

Yerberos (or hierberos) prescribe herbal teas, baths, and poultices to cure physical and mental illnesses. Espiritualistas apply faith, spirituality, and rituals to mend the soul. Hueseros are known for addressing muscle pulls, sprains, and resetting broken bones. While these types may have some overlap, these definitions are useful for understanding THs and the roles they play in the health of the Latine community to preserve health and cure illness.

A new recognition of healing through indigenous practices is spreading in the United States, and the use of alternative and complementary medicine is on the rise.^17^ According to the National Institutes of Health (NIH), alternative and complementary medicine is defined as “use health care approaches that are not typically part of conventional medical care or that may have origins outside of usual Western practice,” when these practices are used in addition to biomedical care they are considered “complementary.”^18,19^

Given evidence that Latine individuals experience limited access to biomedical health care, and the ability of THs to provide care that is linguistically and culturally appropriate, as well as the increasing interest in traditional forms of healing and wellness, the aims of this systematic review were to assess: (1) the prevalence of TH use and types/modalities used by U.S. Latine individuals; (2) health conditions/illnesses, for which TH care was sought; (3) reasons for TH use; and (4) extent of TH use and dual use, which is the use of both TH and biomedical health care.

## Methods

**FIG. 1.**
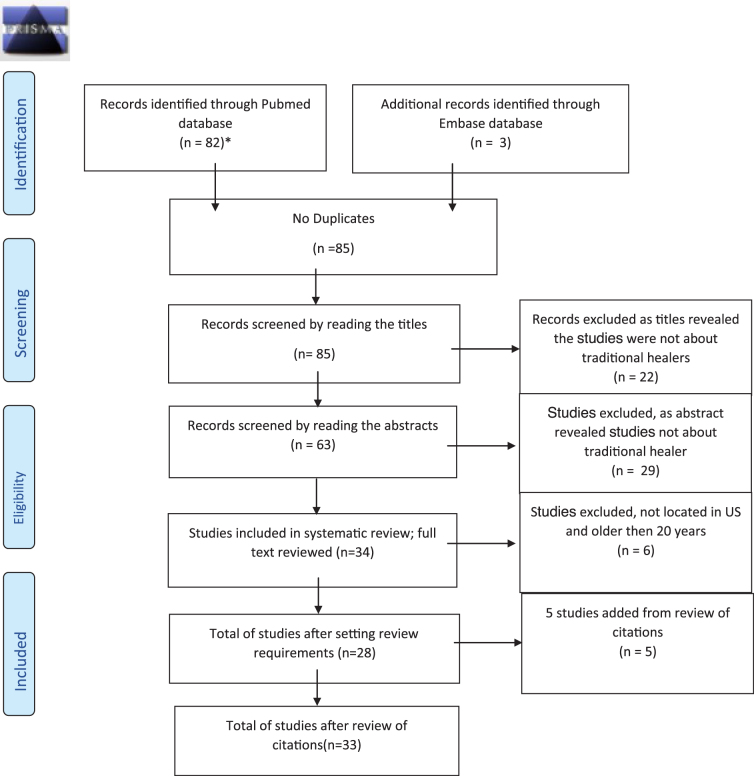
Preferred Reporting Items for Systematic Reviews and Meta-Analyses flow diagram of THs as health care providers for the Latine community in the United States. THs, traditional healers.

This systematic review of the literature was undertaken following the Preferred Reporting Items for Systematic Reviews and Meta-Analyses criteria (Fig. 1).^20^ In October 2020, potential studies were identified through a search of PubMed and EMBASE using the following terms: [“sobador” OR “sobadores” OR “curandero” OR “curanderos” OR “Curanderismo”] and [“traditional medicine”/exp OR “traditional medicine” OR “herbal medicine”/exp OR “herbal medicine”) AND (“curandero” OR “curanderos” OR “curanderas” OR “shaman”/exp OR “shaman”)]. This search was guided by an information specialist at the University of California San Francisco (UCSF) Kalmanovitz Medical Library. Titles that were not relevant were excluded. Studies were included if they fulfilled the following criteria: (1) touched on one of the four TH types of interest; (2) focused on services for the Latine community in the United States, (3) published in English and (4) published between 2000 and 2020. We felt that there may be greater generalizability and relevance to the current health care context if studies were conducted after 2000.

Additional studies were found through citations and an updated PubMed, PsychLit, and CINAHL search in October 2020, using the same search strategy and terms suggested by the research librarian, but expanding to include these additional databases based on research librarian recommendation. Full-text studies of selected articles were read by the authors (E.A., S.C., M.L.C., and K.M.) and coded using an extraction form (Google spreadsheet).

The quality of eligible articles was assessed based on select outcome measures recommended by the Meta-Analysis of Observational Studies in the Epidemiology group,^21^ and included study quality, defined as the representativeness of the sample, the suitability of the measures used, and completeness of reporting. There was not a focus on a specific health outcome (such as diabetes) as THs treat conditions holistically. The following data items were collected on each study: year, title, authors, journal, type of healer, population group, number of participants, documentation/citizenship, geographic area covered, age range, health care access, education, and prevalence of TH use. We calculated prevalence of TH use in each study according to the formula, number of participants who reported TH use divided by the total number of participants included in the study.

## Results

### Characteristics of studies and study participants

Originally, we identified 82 studies using PubMed and 3 from EMBASE. All titles were screened (*n*=85); 57 were eliminated. Full-text review of 28 identified 5 additional studies, which yielded a total of 33 studies that were included in the review (Fig. 1). Of the studies reviewed, 8 were quantitative, 8 were mixed methods, 10 were qualitative, 4 were narrative and 3 were case reports^9,10,13,15,16,19,22–39^ (Table 1). All studies were deemed to be of high or moderate study quality, based on representativeness of the sample, the suitability of the measures used, and completeness of reporting. Characteristics of the individuals in these studies were typically adults, mostly men, and only one study presented a case of a child. Mexico was the most frequent country of origin of the recipients of traditional healing in nine of the identified studies. Included studies had limited reporting of study participant characteristics, and for the most part did not address important characteristics such as educational levels, employment, income, country of origin, or health conditions (Appendix A1).

**Table 1.** Studies included in Traditional Healers as Health Care Providers for the Latine Community in the United States

Study	Year	Study design	Type of healer	Location	Population	No. of participants	Mean age (SD if given)	Education level [mean (SD)]	Prevalence of TH use	Prevalence of conventional medicine use	Dual use conventional +TH use	Health outcome
Reyes-Ortiz^16^	2009	Cross-sectional quantitative	Espiritualistas	United States	Latino	3728	18–49 and 50+	12+ year of education: 54.4%	6% lifetime	NA	NA	Diabetes and depression
Villa-Caballero^22^	2010	Cross-sectional quantitative	Healer (medicine man or woman)	San Diego, CA; Amarillo, TX; Hilo, HI; Raleigh, NC; Des Moines, IA; and Santa Rosa, CA	Latino patients with diabetes	806	56.8 (±0.44) <30, >60	HS or less 33.2%, college: 27.9%, university degree: 31.2%, other 7.8%	9% lifetime	96.2% saw a physician regularly	NA	Diabetes
Lopez^23^	2005	Cross-sectional quantitative	Curandero, Cobador, Yerbero, Espiritualista	Southern California	Mexican American Women	70	28.8 (±7.61)	Undergrad or graduate in the social work program	NA	NA	NA	Empacho (pseudointestinal obstruction), mal aires or mal de aire (or bad airs), mal ojo (or evil eye), embrujo (or supernatural hex), Envidia (intense jealousy), Susto (fright), Espanto (soul lost)
Arcury et al.^37^	2016	Cross-sectional quantitative	THs	North Carolina	Latino/a Farmworkers and Mexican immigrant non-farmworkers	200	Over 18 years old Under 30 or 50 and above	0–6; 45 participants, 7–11; 44 participants, post-high school 11 participants	64% in lifetime, 16% in the past year	NA		Work-related injury
Lindberg^25^	2013	Cross-sectional survey	Curandero/a, Sobador/a, Yerbero/a, Espiritualista	Portland, Oregon	Obese Mexican American women	31	38 (±11.7) Range 19–78	0–6: 11 participants had as, 7–11: 11 participants, high school or attended some college: 9 participants	67.7% lifetime	NA	NA	Weight loss
Kesler^19^	2015	Cross-sectional study	Curandero/a	New Mexico	Latino/a's	NA	NA	NA	NA	NA	NA	NA
Salazar^26^	2013	Cross-sectional study	Curandero/a	United States	Curandero/a-who serve Mexicans and Mexican Americans	NA	57.23 (±14.42)	Traditional healing training	NA	NA	NA	Bilis (bile), Empacho (pseudointestinal obstruction), Encono (festering wounds), Latido (nervous stomach), Mal aire (bad air), Mal ojo (bad eye), Mollera caida (fallen fontanel), Nervios (nerves), Susto (fright)
Padilla^13^	2001	Quantitative surveys	Curandero/a	Colorado	Latino (Hispanic)	405	35.7 (±13.8)	0–6; 20 participants, 7–9; 29 participants, 10–12; 42 participants, post-high school; 27 participants	29.1% in lifetime	83.5% patient of outpatient primary and urgent care clinics	NA	Headache, Empacho (pseudointestinal obstruction), Nervios (nerves), Susto (fright), Embrujado (witchcraft), Mal aire (bad air), Fatigue, Abdominal pain, Back pain, Kidney problems, Encono (festering wound), Melachio (melancholy), Need for cleansing, Diabetes mellitus, Mal ojo (bad eye)
Sánchez^27^	2014	Mixed methods questionnaire	Hierberos/as, Sobadores/as, Sanadores/as, and Suranderos/as	South Florida	Latino Migrant workers, El Salvador, Nicaragua, and Dominican Republic	278	37	27.7% had no formal education, 68.7% reported some formal education, 3.6% reported at least a high school diploma or GED	NA	38.1% in the last 12 months	NA	Work-related injury
Viladrich^28^	2007	Mixed methods	THs	New York	Argentine immigrants	50	NA	Most participants had a primary or high school education, and a few had a college degree from Argentina	NA	NA	NA	Spiritual conditions
Andrews^29^	2013	Mixed methods	Sobador/and Curandero/a	Washington	Latino (Hispanic) Immigrant and migrant families	36	All ages	3 or less: 3, 4–6 years: 12, 7–10 year: 12, 11+ years: 6	60% for treatment of childhood diarrhea	40% of the families noted that they had taken their children to a biomedical facility for diarrhea		Childhood diarrhea
Graham^15^	2016	Mixed methods	Sobador/a	North Carolina	Sobador/a who were born in Mexico	Three sobadores allowed the video recording of eight patient treatment sessions	Young and middle-aged adults	Little to no education-learned from family how to preform healing and one sobador has little college education	NA	NA	NA	Musculoskeletal pain
Ransford^30^	2010	Mixed methods interviews	Curandero/a, Espiritualistas	Los Angeles, California	Latino/a immigrants	96	NA	NA	NA	NA	NA	NA
Shelley^31^	2009	Mixed methods	General TH	New Mexico	Latino (Hispanic) and American Indian	93	All ages	NA	NA	53% patients of primary care clinics	NA	NA
Rogers^32^	2010	Mixed-methods cross-sectional interviews	Curanderos and Yerberos	Oregon	Mexican and Mexican Americans	31	54–85 years (*M*=68)	NA	NA	NA	NA	NA
Macias^33^	2000	Mixed methods	Folk healer	Lennox, California	Latino/a's	Population: 70 participants total but 68 completed survey	33 (±10.2)	NA	7.2% in the past year	46% in the last 12 months	NA	NA
Hunt^34^	2000	Cross-sectional qualitative study	Yerbero/a, Espiritualistas	San Antonio and Laredo, Texas	Low-Income Mexican Americans with type 2 diabetes	43	Mean age: 53.9, range from 29 to 69	1–14 years of education and a average of 8.1 years of schooling	15% lifetime	NA	NA	Diabetes
McCullagh^35^	2015	Cross-sectional qualitative study	Sobador	Southeastern Michigan	Latino (Hispanic) Migrant seasonal farmworkers	6	23–33	NA	NA	NA	NA	Work-related injury
Poss^36^	2003	Cross-sectional qualitative study	Yerbero/a	El Paso, Texas	Mexican Americans	22 (18 females and 4 males)	Mean age: 53, range from 29 to 77	Minimal formal education or were functionally illiterate	NA	NA	NA	Diabetes
Arcury^24^	2019	Cross-sectional qualitative study	THs	North Carolina	Mexican Farmworkers	200	At least 18 years of age	Elementary or less 6 participants, more than elementary 131 participants	28% in the last 12 months	63% in the last 12 months		Acute conditions
Shedlin^38^	2013	Cross-sectional qualitative study	Yerbero/a	El Paso, Texas	Mexican-origin persons living with HIV	113	46	Ranged from 7th to 12th grade (36%) and some have college degree, GED, technical education (16%)	NA	NA	NA	HIV
Hoskins and Padrón^10^	2018	Cross-sectional qualitative study	Curandero/a	California	Curandero/a-who serve immigrant Latino from Mexico and Central America	8 self-identified Curan- dera/os	48–75 years	Practiced for more than 5 years; s/he must have learned the trade directly from another Curandera/o	NA	NA	NA	Spiritual conditions
Quandt et al.^9^	2017	Cross-sectional qualitative study	Sobador/a	North Carolina	Sobador/a from Mexico	40–85 with median age of 55	55	1 participant no formal education, 3 participants completed elementary school, 2 participants completed high school; 1 participant completed 1 year of college	NA	NA	NA	Musculoskeletal pain
Moñoz^47^	2013	Qualitative	THs	Tijuana and San Diego	Health care providers who serve Latino people with HIV	19	Mean (SD): 45 (±9) Mean age 45 years (range 27–59)	Higher medical education	NA	NA	NA	HIV
Sanchez^44^	2018	Qualitative	Curandero/a	United States	Latino (Hispanic)	NA	NA	NA	NA	NA	NA	As susto (fright or soul loss), mal de ojo (evil eye), nervios (emotional distress), envidia (envy), sentimentos fuertes (strong feelings), brujeria (illness caused by witchcraft or sorcery), falta de fe (lack of faith), Susto (fright), Mal de ojo (evil eye)
Sandberg^14^	2018	Qualitative	Sobador/a	North Carolina	Mexican immigrants	24	18 years old or older	Grades 0–6: 10 participants, grades 7–11: 9 participants, grades 12 or higher: 5 participants	100% in the previous 2 years	60%	NA	Musculoskeletal pain
Sorrell^48^	2020	Narrative	Yerberos, Sobadores, Curandero/a,	United States	Latino/a immigrants	NA	NA	NA	NA	NA	NA	Opioid use
Rosario^42^	2014	Narrative	Santeria, Espiritualistas	United States	Latino/a's living with cancer	NA	18 and older	NA	NA	NA	NA	Cancer
Ortiz^49^	2007	Narrative	Curanderos and Santeros	United States	Latino (Latino and Hispanic)	NA	NA	NA	NA	NA	NA	Diabetes mellitus, asthma, and hypertension
Garcia et al.^46^	2020	Narrative	Curandero/a	United States	Mexican immigrants	NA	24-hour groups (anexos): mean age is 40.5 ranging from 20 to 75 years	NA	NA	NA	NA	Substance use
Alarćon R^39^	2014	Case study	Curandero/a Espiritualistas	New York	Latino man with major depressive disorder and anxious distress	1	21	Up to college sophomore	He thought of talking with a friend, a priest, a teacher, or his mother, but ultimately, he decided to see a curandero (healer)	Saw a psychiatrist that prescribed amitriptyline and saw a counseling psychologist. Did not continue because he could not afford it	NA	Major depressive disorder
DeBellonia^52^	2009	Case study	Curandero/a	United States	Latina child	1	4	NA	A 4-year-old female Mexican child was diagnosed by a curandero with espanto	Taken to the Emergency Department after child was unresponsive	NA	Isopropyl toxicity childhood diarrhea
Kennedy^41^	2016	Case study	Curandero/a	Denver, Colorado	Latino male	1	53	NA	A 53-year-old Hispanic male, with chronic low back pain treated by a Curandero	Utility of complementary and alternative medicine in mainstream primary care	NA	Chronic pain, musculoskeletal pain

GED, General Education Development; HS, High School; NA, not available; SD, standard deviation; TH, traditional healer.

**FIG. 2.**
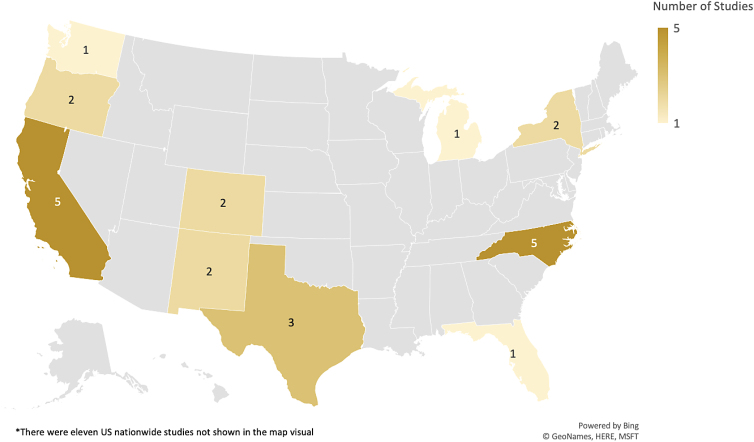
Location of studies for the THs as health care providers for the Latine community in the United States systematic review.

THs in the United States were studied in several key states. Of the 33 studies in this systematic review, 9 were national studies. Of the remaining 24 studies, participants resided in California (5), North Carolina (5), Texas (3), Colorado (2), New Mexico (2), New York (2), Oregon (2), Florida (1), Michigan (1), and Washington (1) (Fig. 2).

### TH terms and definitions

General TH terms used and their definitions, common TH subtypes, and the locations of practice are found in Table 2. The most common type of THs were curanderos (overarching terms that included subtypes), followed by sobadores, espirtualistas, and yerberos.

**Table 2.** Traditional Healer Practitioners, Subtypes and Types of Treatment Administered

TH type	Subtype	Definition	Physical manipulation and treatment	Mental health treatment	Use of herbal therapy	Spiritual or religious practices
Curanderos (healer/general)		Use diverse healing approaches that include physical treatments, psychic healing, spiritual healing, and spiritualism	Yes	Yes	Yes	Yes
	Hueseros	Set broken bones, treat sprains, and muscle pulls	Yes			
	Yerberos	Prescribe herbal teas, baths, or poultices to cure physical and mental illnesses		Yes	Yes	Yes
	Sobadores	Use massage, mobilization, and manipulation to care for pulled muscles and injured joints, as well as moving internal organs	Yes			
	Espiritualistas	Faith healers who pursue to heal the soul				Yes
	Botánicas	Herbal pharmacy dispensing dried herbs and tinctures, as well as religious/sacred items				
	Santerías	Multiuse locations; they are thought to hold supernatural forces, can be used to mobilize these beneficial supernatural forces, and can overall decrease uncertainty and stress				

### Prevalence of TH use, and types/modalities used

The use of THs among the U.S. Latine community is common, possibly as common, or more common than use of biomedical health care providers. In a recent studies of 200 Latine individuals, 64% used THs.^24,37^ Among these, 19.5% had been treated by a sobador, 4.5% by a curandero, 2.0% by an herbalist, and 2.0% by a spiritual healer.^24^ In another study of 806 participants, nearly 60% of Latine individuals described using folk healing methods and/or healers for the treatment of their children's diarrhea.^29^ In Mexican women, the lifetime prevalence is as high as 67.7%.^25^ A study with 405 subjects, found that 118 (29.1%) had been to a curandero at some time in their lives and 91.3% of participants knew of curanderos.^13^ The use of a “medicine man or woman” was more than three times higher in Latine individuals and Native Americans compared with Whites.^22^ From the studies reviewed, the lifetime prevalence of TH use varied from 6% to 67.7%, and the prevalence in the last year varied from 7.2% to 28%, partially depending on the sociodemographic differences among the Latine individuals in these studies (Table 1).

THs practiced a range of treatment modalities to address these conditions (Supplementary Table S1). These modalities can use physical treatments like rubbing or massaging.^10,15,25,40,41^ *Santerias* are commonly used by THs and function as the visible door to the invisible world of folk healing.^42^ *Yerberos* use *botánicas* as a resource for people who seek ethnomedicine and ethnobotany alternative medicine, which contains naturopathic treatments involving herbal teas and foods.^29,43^ For example people with diabetes use a concoction of herbs as a supplement in addition to their medication^14,32,37^

THs diagnose clients based on the client's description of their current ailment and with minimal history, compared with biomedical health care. THs let the client talk about their life events because this relaxes them, but, do not rely upon laboratory values to heal.^9^

Curanderos use a practice of ancient holistic treatments that heal individuals through targeting three hierarchical realms: the religious and/or spiritual realm, the emotional realm, and the process of health and mental illness.^10^ Connection with the natural and supernatural, finding emotional and mental balance, and the holistic/naturopathic alternatives to release endorphins and decrease cortisol to ease pain and stress.^10^ Some curanderos view physical health as dependent on an individual's four bodily fluids (humors), which consist of hot fluid of the blood, yellow bile, cold fluid of phlegm, and black bile.^34^ Some used spiritual healing practices based on a belief that healers were gifted higher powers to heal by God. Their practice is not identified as a career choice but a mission.^9^

Prayer is used to bring healing powers to medicine.^34^ The idea of spiritual healing originates from indigenous communities residing in present-day Mexico and Central America. These indigenous communities had a belief, stated in Lak'ech, an indigenous language, *tú eres mi otro yo* (“we are one”). It states “we,” the people, are one within a spiritual connection to everything surrounding them—family, community, the elements, stars, moon, sun, and universe.^10^ Spiritualism is the connection with the mind, body, and spirit. THs recognize that the spirit is responsible for the balance and restoration of health.^10^ People treated by THs explain that when the human–spirit connection encounters an adverse experience, the body and spirit are at greater risk of being out of balance within themselves, others, and/or the environment.^10^ Spiritual healers believe illness is caused by this imbalance and they act as mediums to heal the imbalance.^41^

THs also call and conjure spirits to assist people with their healing process (spirits of deceased relatives, or spirits of famous THs).^26^ Religion additionally could play a role in the practice of spiritual healing. Some THs use practices from Catholicism for example pictures, statues, saint medallions, crosses, holy water, candles, altars, and prayer.^13,44^ Additionally, those who go to THs also use prayer as a primary form of self-care^24^ because it may reduce clients' levels of stress and anxiety.^30,34^ A national qualitative study found that 6% of participants consulted a curandero, 60% prayed for healing, 49% asked others to pray for healing, and 69% considered spiritual healing as very important.^16^

### Health conditions addressed

Table 1 describes various conditions identified across a spectrum of physical, mental, and spiritual health, for which the Latine community sought THs. THs were sought out to address conditions related to gastrointestinal problems, empacho (pseudointestinal obstruction), abdominal pain,^13,45^ childhood diarrhea,^10,29^ diabetes mellitus,^13,16,22,34,36^ musculoskeletal pain^9,14,15,39,41^ and injuries,^35,37^ insomnia,^41^ weight loss,^25^ substance use,^46^ headache,^13^ nervios (nerves),^13^ Susto (fright), mal aire (bad air),^13^ back pain,^13^ kidney problems,^13^ melachio (melancholy),^13^ mal ojo (bad eye),^13^ spiritual conditions,^10,28^ HIV,^38,47^ cancer,^41^ and depression.^16^ In farmworkers, the majority (82.5%) sought THs to help with acute conditions (7.5% used them to treat chronic conditions),^24^ primarily work-related injuries that they viewed as a consequence of hard physical labor.^35^ Supplementary Table S1 depicts some of the treatment modalities for these conditions.

### Reasons for use of THs

Reasons for the use of THs among Latine individuals included accessibility/convenience, affordability, and acceptability due to cultural and language congruence. Widespread structural and cultural factors affecting the Latine community in the United States were evident in their decision making related to the use of THs, including immigration status, dissatisfaction with biomedical medication, folk illnesses and cultural healing practices, and lack of familiarity on the part of biomedical health care practitioners with these cultural viewpoints of wellness and illness.^16^

#### Accessibility/convenience

THs operate from small multiuse spaces embedded in the community or in settings in nature, making them highly accessible. THs' homes are typically located in the Latine communities they serve.^13^ They offer their services until late evening, so they are accessible to people who work long hours. Sometimes, THs work out of *botánicas*—herbal pharmacies dispensing dried herbs and tinctures, as well as religious/sacred items. Botánicas can be part of a community market, a local flea market, or located in a sacred private natural location. THs also work from *santerías*. Similar to botánicas, santerías are often found in community multiuse locations where candles, books, oils, herbs, sacred necklaces, and medicines are sold. Santerías also offer spiritual consultations or rituals that are thought to mobilize beneficial life forces to decrease uncertainty and/or stress. Visiting a santería can help to maintain or reinstate balance and a sense of control over one's life, with the goal being to live the life that has been assigned to you in the best way possible.

THs are integrated into their communities, taking on many roles (e.g., doctor, psychiatrist, religious guide). They may address physical, emotional, and spiritual needs of the client.^26^ Furthermore, as stated in one study, “a traditional healer's higher purpose is to tend to the spirit of people.”^10^ They do this by giving hope to clients and by building their emotional support network. Hope grows because the TH reassures them that the future will be better than the present, especially in those who are going through life transitions. THs also provide safe spaces for people who have failed to find biomedical solutions for their problems, by listening to people's worries and uncertainties.^28^

#### Affordability

Community members rely on THs to provide affordable services and treatments. This may be even more pertinent for Latine individuals who are undocumented or otherwise ineligible for employment- or government-based health insurance. One participant reported that “as a person without health insurance it is easier, safer, and cheaper to go to a traditional healer than a [biomedical] clinician.”^27^ Participants understand that it is necessary to seek medical care but the high cost is the most frequently cited barrier.^33^ A study found that participants believed THs are accessible and affordable, unlike biomedical health care services.^32^ Many Latine individuals work in low-wage jobs that do not offer health insurance, such as farming or domestic work and therefore cannot afford biomedical health care treatment.^35^ Many Latine individuals are exploited for labor and stripped of workers' rights, for example in the construction industry if employees report a work-related injury they risk getting fired so they would rather go to a TH.^24^

The cost of treatment varies by TH. A study interviewing THs found that one had a set fee, $30 for a three-visit course of treatment, whereas a sobador considered $50 reasonable since massaging takes a toll on their hands. THs set affordable prices as they see their job as a calling to provide a community service. Some sobadores leave it up to the client based on their ability to pay the sobador(a), with clients paying between $1 and $100.^9^

#### Acceptability due to cultural and linguistic congruence

Traditional and complementary services, such as THs, can be more acceptable to some members of the Latine community because these culturally competent and sensitive approaches can improve health outcomes, decrease stigma, and address disparities in access to treatments.^48^ Barriers to biomedical health care identified were high cost of services, distance to providers, lack of transportation, and limited hours of availability of services, all of which contribute to participants' frequent use of THs.^35^ Another study found comparable results citing problems with communication, establishing financial eligibility, and extremely long waits for medical appointments.^30^

Latine individuals may favor THs over biomedical providers due to language concordance or the inability to follow medical jargon. One large study found that after seeing a biomedical/medical professional, Latine individuals report confusing language used by providers (23%) or feeling frustrated (27%).^16^ Another study observed that 59% of participants rated their capacity to communicate with their biomedical health care providers through survey as “sometimes have problems communicating” and 6% indicated “frequently had difficulties” with biomedical.^23^ Communication barriers among biomedical physicians and Latine individuals go further than merely not speaking the same language. Even if the biomedical physician speaks Spanish, THs may communicate more effectively due to their sharing the same cultural background.

### Dual use of THs and biomedical health care

None of the studies included systematic classification of health care seeking by Latine individuals as: TH used alone, biomedical care alone, or dual use; therefore, it was not possible to estimate the prevalence of dual use. THs, did however, acknowledge the legitimacy of biomedical diagnoses and medicine.^9^ THs had clear notions as to how to heal specific conditions and recognized that they could not address all biomedical diagnoses. Consumers of TH services expressed that it would be ideal to have a health care provider who understood and was willing to use both traditional and biomedical systems to treat their illness.^36^ One study found that patients are open to discussing their use of THs with their biomedical health care providers if they have an accepting and nonjudgmental attitude, while not making patients feel rebuked or fearful.^31^ One study concluded that understanding the use of traditional healing remedies could improve approaches to biomedical health care because these professionals may benefit from positive applications of common TH practices.^19^

## Discussion

This systematic review, covering the past 20 years, demonstrates that THs are commonly used by the Latine community living in the U.S. Based on the studies reviewed, the use of THs among Latine individuals in the U.S. ranges from 6% to 67%. Participants in these studies tended to be adults, male, and Mexican American in origin. Latine individuals sought THs for common conditions, such as gastrointestinal problems, childhood diarrhea, diabetes,^30^ musculoskeletal pain and injuries, insomnia, fever, weight loss, substance use, and emotional distress. Conditions included those that disproportionately affect Latine individuals, for example diabetes. THs or *curanderos* were often generalists or in some instances specialists like sobadores, yerberos, espirtualistas, and hueseros. The Latine community sought the services of THs for reasons of accessibility/convenience, affordability, and cultural and linguistic congruence.

Our estimates of the prevalence of TH use among Latine individuals in the United States are consistent with two prior reviews that reported high use of complementary alternative medicine.^49^ Another prior review found that traditional healing practices among Mexican and Mexican Americans may be as high as 75% in some parts of the United States.^50^ Latine individuals may seek care from THs before accessing the biomedical health care system. Although it is not stated explicitly in most studies, Latine individuals may have been left with no other option than to use THs as their only health service if they were unable to access biomedical/medical systems. As such, understanding the role that THs play and developing better reciprocal relationships between traditional and formal health care may lead to increased health equity.

THs are so widely used because they address widespread barriers to use of the biomedical health care system among Latine individuals. Because THs live in the same communities as their clients and they offer evening hours, they alleviate barriers of missed work and lack of transportation. These reasons for use are concordant with a prior systematic review.^12^ They tend to be less costly than biomedical health care and are culturally and language concordant. Moreover, there may be increased trust or confidence in THs because they do not ask about immigration status. Their care is viewed as more person-centered because they use a mix of spiritual and religious practices, which are familiar and treat the “whole person.” Given the high prevalence of use and other advantages, understanding how THs establish trust and meet the needs of the Latine community may help inform how to address some of these common barriers in biomedical/medical contexts.

Despite the high prevalence of TH practices, there is a dearth of peer-reviewed literature on the topic. Ongoing and future studies can help elucidate the optimal use of THs to serve the needs of the Latine community. Future research should examine whether Latine individuals utilize THs as the first point of care and whether they choose to use TH out of preference or because it is the only type of care that is feasible for them. Future efficacy and effectiveness research is needed to ascertain when TH services are beneficial (specific treatment modalities and diseases or conditions), as well as any potential risks or harms. Trials could test whether TH modalities when combined with biomedical health care (dual use) yields better health outcomes than biomedical health care or TH use alone. Qualitative studies with THs may elucidate where the healers feel they are making an impact, how they view their role in the public health/medical system and their relationship to biomedical/medical health systems. For example, lay health care workers or *promotores/as de salud*, traditionally play a critical role in bridging access of Latine communities to biomedical approaches.^51^ This type of model could be expanded to include THs.

Several limitations of this article deserve comment. The populations tended to not be well characterized in terms of education, income, languages, language proficiency, and health conditions. Therefore, generalizability of our findings to specific Latine individuals or specific health outcomes is difficult to assess. There were a range of ages included in our studies; most of the groups had low formal education. It is unclear whether any generational or educational differences were the reasons for TH use. Recognizing that Latine communities are heterogeneous and diverse, we encourage future studies to report additional ethnic subgroup details and focus on the health outcomes of those served by THs. While we were unable to assess the effectiveness of traditional therapies in treating prevalent disease conditions, our findings provide valuable insight into the heterogeneity in the disease conditions and treatment approaches addressed by THs. This review focused on the U.S. Latine community and excluded those from other countries, thereby missing knowledge external to the United States.

Similarly, studies published in Spanish were not incorporated. A possible source of bias is publication bias, that is, researchers with data on the prevalence of TH use in diverse race/ethnic groups did not publish their data, therefore, our review may underestimate the prevalence of use. Furthermore, there is great variability in the definitions used for the various types of THs that were included in this study. However, a strength of our review is that we provide a detailed definition of several key TH types, modalities, and conditions/illnesses for which they are used.

There have been a few past reviews focused on curanderismo and healing practices in the United States. These studies did not cover 20 years, nor did they extract information on the use of THs alone, or dual use with the biomedical/medical system. Our review expands and improves on this prior work by using a systematic approach guided by a library scientist and covers 33 studies assessed for their quality in the peer-reviewed public health literature spanning 20 years. Instead of framing the use of an indigenous, nonbiomedical health practice as an alternative, or “less good than biomedical healthcare,” we took the approach to learn about the community and their needs on “equal footing.”^10^

Public health practitioners and physicians in the United States may benefit from understanding the traditional forms of healing used in Latine communities. Seeking care from THs should be further studied and utilized to optimize care. As is the case for biomedical health care, both the potentially beneficial and iatrogenic effects of treatments must be systematically studied, as harmful side effects and toxicities associated with TH practices have been known to occur.^52^ Knowledge exchange between THs and physicians could be mutually beneficial and help address potential treatment interactions and iatrogenic effects. THs could work alongside public health and medical professionals to educate them on cultural humility and cultural relevance, thereby perhaps engendering more trust in the biomedical/medical system as cited by one study highlighting this pairing as a beneficial partnership.^48^

Biomedical/medical institutions could implement additional services (such as linkages to traditional healing) to be able to improve the health of Latine and other communities that seek care from THs. Indeed, younger doctors seem open to this idea. At the University of New Mexico Public Health and General Preventive Medicine Residency Program, all residents agreed that: “not knowing about traditional healing practices in use by patients could result in adverse outcomes due to interactions of biomedical/medical treatment and substances such as herbs used by the patient” and they “would recommend learning about traditional healing practices to other health care providers.”^19^

## Conclusions

THs are commonly used by Latine individuals residing in the U.S. THs deliver person-centered care and address systemic barriers faced by Latine communities in the United States by providing linguistic and culturally concordant care that is accessible, affordable, and incorporates spiritual, physical, and mental healing. Coordination of THs with biomedical health care represents an opportunity to improve the delivery of patient-centered, high-quality care for the Latine community and improve health equity.

## Supplementary Material

Supplemental data

## Abbreviations Used

NIH: National Institutes of Health
SD: standard deviation
THs: traditional healers
UCSF: University of California San Francisco

## Appendix A1.

1. Search #1: PubMed Search on [Date] using:

- Keywords: sobador OR sobadores OR curandero OR curanderos OR Curanderismo
- Search #2: Embase search on [Date] using:
- “traditional medicine”/exp OR “traditional medicine” OR “herbal medicine”/exp OR “herbal medicine”) AND (curandero OR curanderos OR curanderas OR “shaman”/exp OR shaman
