# Supplementary material for: Traditional Healers as Health Care Providers for the Latine Community in the United States, a Systematic Review
Source: Health Equity. 2022 Jun 15;6(1):412–26. doi: 10.1089/heq.2021.0099 (PMC9257545; doi:10.1089/heq.2021.0099)
Supplement: Supplemental data [file Suppl_TableS1.docx]

| **Supplemental table 1. Conditions, major treatment modality and herbs or plants used** | | | | |
| --- | --- | --- | --- | --- |
| **Condition** |  | | **Major treatment modality**(45) | **Herbs and plants.** (34) |
| **Overall Health** | | **Balancing of the four humors** |  |  |
| **Physical Conditions** | | |  |  |
|  | | Gastrointestinal illness: an imbalance in metabolism results in gastrointestinal issues, called *empacho*. Strong foods such as dairy products, pork, and eggs may cause imbalance**.**(23) | Gastrological supporting herbs are used to treat abdominal pain and gastrointestinal issues that are associated with pregnancy. (23) | *Yerbabuena* (peppermint) and *manzanilla* (chamomile) |
|  | | Diabetes | Traditional healers prescribe individuals with diabetes and high blood sugar/pressure with *nopal* (cactus leaf), because of the belief that it can purify the blood. | *Nopal* (cactus leaf) is frequently consumed by boiling and mixing the leaf with oils and additional herbs for salads and soups or by blending it with fruits and vegetables as a smoothie for breakfast. (36) Furthermore, traditional healers aid agricultural laborers with herbs such as *brickellia* (brickle bush), campanas *amarillas* (yellow bells), *lagrimas de San Pedro* (Job’s tears), prickly cactus, and lemon juice to prevent and control diabetes. (24) |
|  | | Muscle pulls | *Sobadas*(massages): Massages, known as *sobadas*, are a therapeutic touch. *Sobadas* release endorphins that help counter cortisol (a stress hormone), which are useful for calming the body. |  |
|  | | Insomnia | *Herbally infused teas* are utilized by curanderos to treat insomnia and alleviate fevers(23) | *Tea made from flor de tila* (linden blossoms) |
|  | | Minor fever | *borraja* (borage) (23) |  |
|  | | Weight loss | Herbal teas are consumed to limit fat absorption. | Herbally infused teas from *agracejo rastrero* (Barberry, Evolvus Hummularius), *Arenaria* (Sand plantain, plantago arenaria), and *Carqueja* (Baccharis genistelloides). (25) Other home remedies consisted of rubbing olive oil and sea salt oil on their stomachs and consumed water with lime juice and vinegar before breakfast and to sleep with a band wrapped around their stomach (25). |
|  | | HIV/Cancer side effects | help with for emotional support, support general health and their immune systems, (38, 46) |  |
| **Substance use/abuse** | | Alcohol addiction | *General Detoxification*: This treatment is meant to help the body with detoxification. | Saline solutions, vitamin regimens, and herbal remedies are all used daily to cleanse the liver of any toxins and create a clear state of mind for the individual.(45) |
|  | | Opioid addiction | Mexican traditional medicine 13 doors technique (47) | Combination of curanderismo treatment and biomedical treatment if have additional chronic pain. (40)  TH assisted with appointments, massages (*massajes*), and spirit/energy activities (47) |
| **Spiritual conditions** | |  |  |  |
|  | | Functioning spirit provides good mental health, but dysfunction in the soul can cause sadness, rage, and envy | The goal is to remove the illness and bring the spirit back to a balanced state (10). *Temescal*(Sweat Lodge): The sweat lodge, known as *temescal*, is a sacred place amongst *curanderos.* T*emescal* begins with the individual preparing for the emotional journey they will embark on. Emotional awakening is strongly encouraged for individuals while they are revisiting fears and emotional traumas experienced in their lives while under the guidance of a curandero. The heat and darkness used in a temescal start an emotional release, while the perspiration helps to release all the toxins in the body. Ritual rinsings are performed throughout this journey and sometimes may include prayers for those who seek forgiveness. |  |
|  | | *Espanto* which is the a severe form of fright, an emotional illness caused by an event that traumatizes an individual and causes individual significant distress” (51) |  | *Raspada* is a common form of spiritual healing to align and correct the spiritual energy practiced amongst traditional healers (40). |
|  | | Strong emotional distress | *Limpias*(Spiritual Cleansings): *Limpias* are spiritual cleansing rituals conducted to restore an emotional balance in an individual who suffers from traumas, despairs, and fears. *Limpias*are a form of holistic care used to cleanse the mind, body, and spirit. There are different forms of *limpias*, but many involve “an egg used to absorb and remove destructive and negative energies; it may involve brushing the body with fragrant herbal brushes, fire or water for this cleansing treatment”.(48) | Many curanderos also perform *limpias*, which is a spiritual cleanse by using a handful of herbs and running/sweeping it across the whole body while chanting. |
|  | | Emotional trauma that has risen in their life. | *Pláticas*(Counseling):  confidential talks between the individual seeking help and the *curandero*.  Some *pláticas* are for family members of the individual who is seeking help because many family members usually experience distress when a loved one is ill. |  |
|  | | *Mal de ojo*’ or evil-eye | Prayer is used to help heal an individual. Intercession is practiced amongst *curanderos* for them to pray on behalf of the individual in their presence. |  |
|  | | *Mal de aires* is a cultural belief that specific wind drafts can create illnesses. | Hot drafts can induce digestive issues, pregnancy issues, sore throat, rashes, and kidney ailments, while cold drafts develop illnesses related to colds, tuberculosis, and headaches (23, 34) | *Mal de aires* can be cured using *pirul* (pepper or peppercorn tree) and peppermint leaves; (23, 34)  treated by prescribed teas, herbal baths, or poultices; or by foods that have the opposite temperature. |
